# l‐carnitine downregulate the muscle wasting effect of glucocorticoids in pemphigus patient: A randomized double‐blind placebo‐controlled study

**DOI:** 10.14814/phy2.15657

**Published:** 2023-04-20

**Authors:** Zeinab Noormohammadi, Hana Arghavani, Mohammadhasan Javanbakht, Maryam Daneshpazhooh, Mahmood Jalali

**Affiliations:** ^1^ Department of cellular and molecular Nutrition, School of Nutritional Sciences and Dietetics Tehran University of Medical Sciences (TUMS) Tehran Iran; ^2^ Department of Public Nutrition, School of Nutritional Sciences and Dietetics Tehran University of Medical Sciences (TUMS) Tehran Iran; ^3^ Autoimmune Bullous Diseases Research Center Department of Dermatology team Tehran University of Medical Sciences Tehran Iran

**Keywords:** creatine kinase, IGF‐1, myogenin, myostatin, pemphigus vulgaris

## Abstract

Pemphigus Vulgaris (PV) is a blistering autoimmune disease caused by autoantibodies against desmoglein 1 and 3. Treatment options are limited to corticosteroids and immunosuppressants. The myotoxic effect of glucocorticoids is a fact that has been elucidated. So, the development of efficacious treatment approaches to combat muscle wasting is of great importance. Considering the adverse effect of glucocorticoid therapy in pemphigus patients and altered muscle metabolism, this study aimed to investigate the effect of l‐carnitine supplementation which can be useful in combating muscle‐wasting impact of glucocorticoid therapy. In this randomized double‐blind placebo‐controlled trial 44 pemphigus patients aged from 30 to 65 years, receiving glucocorticoid therapy were selected to evaluate the suitability of l‐carnitine (LC) as an anti‐wasting substance. Patients were randomly divided into two groups to receive 2 g/d l‐carnitine or placebo for 8 weeks; serum markers of muscle metabolism (IGF‐1, creatine kinase, myogenin, myostatin) was evaluated before and after the l‐carnitine supplementation. Paired *T*‐test was used to analyze the differences between variables before and after the intervention. Therefore, the student's *t*‐test was performed to find any differences in baseline characteristics and dietary intakes between the trial groups. LC intake led to a significant rise in serum IGF‐1 and a reduction in CK and myostatin levels compared to baseline (*p* < 0.05) but there were no significant inter‐group differences in IGF‐1 and CK levels; There was also a significant reduction in myostatin level in LC group (*p* < 0/05). Myogenin levels decreased in both LC and placebo groups but the decrease in the placebo group was significant (*p* = 0/008); it means LC prevent the myogenin decreasing trend in the LC group compared to placebo. In conclusion, LC supplementation beneficially changes the level of IGF‐1 and myostatin and improves muscle metabolism and regeneration in PV patients.

## Graphical abstract

Glucocorticoids adverse the normal muscle metabolism and leads to protein breakdown and muscleatrophy through various pathways and L‐carnitine downregulates the muscle catabolism pathways,leads to protein anabolism and muscle growth.

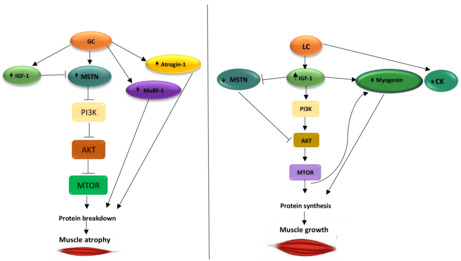

## 1 INTRODUCTION

**FIGURE 1:**
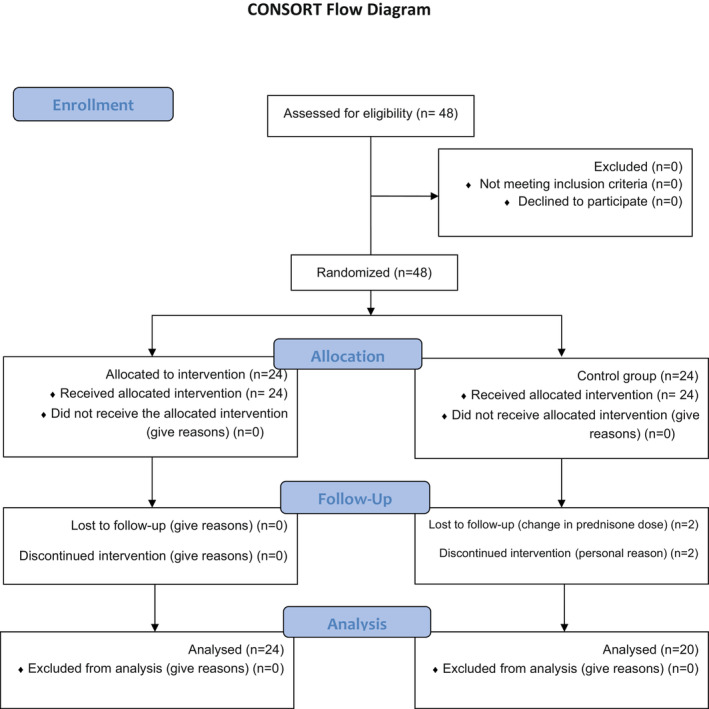
CONSORT Flow Diagram.

Pemphigus vulgaris (PV) is a potentially fatal autoimmune blistering disease that affects the skin and mucous membranes. It is characterized by autoantibodies against the antigens of the intercellular junctions of keratinocytes, desmoglein (Dsg) 1 and 3 (Dănescu et al., 2018) which play a major role in desmosomes cell–cell adhesion between keratinocytes and cause blister formation with acantholysis. Treatment options are limited to corticosteroids and immunosuppressants, which just alleviate the symptomology and provide no cure for patients. On the other hand, long‐term use of such treatments may lead to muscle atrophy. Skeletal muscle atrophy is characterized by a decrease in the size of the muscle fibers. Considering the myotoxic effect of glucocorticoids, it seems that the administration of dietary supplements which decrease muscle‐wasting trend might be useful in managing pemphigus comorbidities (Figure 1).

l‐carnitine (LC) is a water‐soluble quaternary amine that is essential for the normal function of all tissues. LC is primarily derived from dietary sources, especially meat and dairy products, and to a lesser extent endogenously synthesized in the liver and kidney. The main role of LC is being the cofactor for transporting the long‐chain fatty acid to the inner membrane of mitochondria for β‐oxidation (Evans et al., 2017). In addition, LC increases protein biosynthesis (Owen et al., 2001) and suppressed genes responsible for protein degradation in skeletal muscle (Keller et al., 2012). A clinical trial in HIV‐infected patients revealed that LC supplementation results in an improvement in drug‐related myopathy (Vaz & Wanders, 2002). Recently, it has been shown that carnitine downregulates genes involved in the ubiquitin‐proteasome system (UPS) in the muscle of pigs and rats (Keller et al., 2011; Keller et al., 2013) and decreases the muscle RING‐finger protein‐1 (*MuRF1*) that is involved in protein catabolism. Furthermore, another experimental study showed that LC administration reduces proteolysis in skeletal muscle, increases muscle weight, and improves parameters of physical performance in tumor‐bearing rats.

Glucocorticoids are immunosuppressants widely prescribed for a multitude of diseases. With over 50 years of usage, they are among the most recognized mycotoxins agents. The long period of treatment and high dose of these drugs have many adverse effects on patient's physiology such as muscle atrophy, a complication which leads to intolerance to treatment, poor prognosis, low quality of life, and high mortality are some of the side effects in patients (Ringseis et al., 2013). Loss of the muscle mass is result of the imbalance between protein catabolism and protein anabolism; glucocorticoids adverse the normal protein and muscle metabolism, induce apoptosis, and reduce protein synthesis in myocytes and this process leads to muscle atrophy (Ringseis et al., 2013). Therefore, finding new ways to reduce the muscle‐wasting effect of glucocorticoids in PV is required.

## 2 METHODS AND MATERIALS

### 2.1 Subjects

Forty‐four pemphigus patients aged from 30 to 65 years under corticosteroid therapy were chosen from Razi dermatology hospital, Tehran University of Medical Sciences (TUMS), Tehran, Iran to participate in the study. The intervention was continued for 8 weeks. To compute the sample size, we applied a parallel design randomized controlled trial formula based on 0.05 for type one (α) error and 0.20 for type two error (β). The inclusion criteria were: history of pemphigus disease at least for 1 year; using corticosteroid alone or with methotrexate, azathioprine, or mycophenolate mofetil; not having cardiovascular disease, renal, hepatic, and inflammatory disorders; not taking any antioxidant supplements within the past 3 months before the intervention; not taking drugs with obvious interaction with carnitine or influence its metabolism, that is, theophylline or valproate; BMI < 35; being a non‐smoker. The study protocol was approved by the Ethics Committee of Tehran University of Medical Sciences (Iran) and registered the Iranian Registry of Clinical Trials (IRCT code: IRCT2015062322769N4). All subjects were made aware of the content of the study written informed consent which was obtained from each participating subject.

### 2.2 Study design

This was a randomized, double‐blind, placebo‐controlled study. The treatment duration was for 8 weeks. Forty‐four patients with pemphigus followed by Razi hospital accepted to participate in the study. Participants were matched for age and gender and then randomly allocated to receive 2 g LC tartrate per day (*n* = 24) or placebo (*n* = 20), subdivided into two equal doses of 1 g before breakfast and dinner for 8 weeks (l‐carnitine, Asal daroo Kish Pharmaceutical Company in Kish). The control group received a placebo for the same duration and same appearance (Placebo, Asal daroo Kish Pharmaceutical Company in Kish), coded by the company to assurance blinding. Stratified randomization was used to allocate subjects to a main drug and placebo. Researchers were not informed about the randomization process until the completion of data analyses (concealment). As well, during the study, no researchers and patients were aware of the drugs randomly allocated. Randomized allocation participant's group assignment was done by the technician. The participants were asked to keep their usual dietary intake and physical activity and not change their medications. Compliance with the LC supplementation was affirmed via serum LC measurement. Patients were monitored weekly for any possible side effects of l‐carnitine through phone interviews. These data included serum concentration of IGF‐1, myogenin, myostatin, and the activity of creatine kinase enzyme. The IGF‐1, myogenin, and myostatin were measured by kits, and creatine kinase was measured by the auto analyzer device. The body mass index was calculated from body weight and body height.

### 2.3 Clinical assessment

The severity of oral mucosa and skin involvement was measured by a simple scoring system (Harman scores) of 0–3 (0, quiescent; 1, mild; 2, moderate; 3, severe). For curtailing observer bias participants were assessed by a single expert physician. Body weight and height were measured by a digital scale (to the nearest 0.1 kg) and stadiometer (to the nearest 0.1 cm), respectively (Seca). Waist circumference (WC) was assessed with a no‐stretching tape measure (Seca) at the midpoint between the costal margin and the iliac crest with no pressure on the body surface. BMI was computed as weight (kg) divided by the square of height (m^2^). All patient's medical and drug histories were obtained.

### 2.4 Dietary intake, anthropometry, and physical activity assessments

All patients underwent routine physical examinations at the onset of the study; at the beginning and at the end of the intervention period. Body weight was measured to the nearest 0.1 kg using a Seca scale with the patients being barefoot and wearing light clothing. Height was also measured using a mounted tape, with the participants' arms hanging freely by their sides and recorded to the nearest 0.5 cm. BMI was calculated by dividing weight (in kilograms) by the square of height (in meters). For evaluation of physical activity level before and after intervention an international physical activity questionnaire (IPAQ) was completed for each subject by an instructed interviewer (Committee, 2005). Participants were classified into high, moderate, or low physical activity levels based on the grade scoring protocol of the short form of IPAQ. Information on food intake was collected by using 24‐h recall method for 3 days (including 2 working days and 1 weekend day) at the beginning and the end of the trial. The dietary recalls were analyzed with Nutritionist IV software (First Databank) adjusted for Iranian foods. The participants were asked not to change their dietary habits and physical activity during the study.

### 2.5 Biochemical assessment

Data were collected in the morning, after a 12–14 h overnight fasting at the beginning and the end of the trial. Venous blood samples (10 mL) were drawn into tubes containing EDTA or heparin, then immediately centrifuged (Hettich D‐78532) at 3000 r.p.m. for 10 min to separate serum and stored at −80°C until analysis. Serum levels of myogenin, myostatin, and IGF‐1 were determined by enzymatic methods using ELISA kits (Cusabio Ulm) and creatine kinase by auto‐analyzer system (Selectra E, Vitalab). Moreover, Serum LC was measured by an ELISA kit (Cusabio Ulm) and ELISA plate reader (Elx800, Bio‐Tek Instrument Inc.) at a wavelength of 450 nm.

### 2.6 Statistical analysis

Statistical analyses were performed with SPSS version 21 software (SPSS, Inc.), and all parameters were reported as means ± standard deviation, respectively. Means were considered significantly different at *p* < 0.05. Normal distribution of all definite parameters was tested by the Kolmogorov–Smirnov test. Variables not normally distributed were analyzed using Log transformation. Qualitative and normally distributed quantitative variables were displayed as numbers (percentages) and mean ± standard deviation. Categorical and demographic variables were analyzed by the Pearson chi‐square test. The differences between variables before and after intervention were compared by paired *t*‐test. The Student's *t*‐test was performed to find out any differences in baseline characteristics and dietary intakes between the trial groups. We utilized t‐tests for independent samples to compare data from controls with the placebo data of patients. Analysis of covariance (ANCOVA) was used to identify any differences between the two groups at the end of the study, adjusting for baseline values and covariates.

## 3 RESULTS

### 3.1 Participant demography

From a total of 48 subjects who met the inclusion criteria and entered the study, four patients in the placebo group (due to a change in dose of prednisone (*n* = 2) and personal reasons (*n* = 2)) were withdrawn from intervention and lost to follow‐up. Therefore, the data were reported for 44 patients (24 in the l‐carnitine group, and 20 in the placebo group). The analyses were performed according to the ITT approach. Participants did not report any adverse effects with the l‐carnitine consumption or placebo during the study which confirmed the safety of the l‐carnitine supplementation. The age and BMI of the participants were 52.05 ± 6.13 years and 32.02 ± 3.12 kg/m^2^, respectively. As presented in Table 1, there were no significant differences in demographic characteristics, duration, and severity of the disease or in physical activity level between the study groups at baseline.

**TABLE 1:** Baseline characteristics of study subjects^a^.

Variable	l‐carnitine group (*n* = 24)	Placebo group (*n* = 20)	*p*‐value^b^
Male/Female	10/16	10/16	1.00
Age (year)	41.04 ± 9.65	40.65 ± 9.90	0.88
Weight (kg)	77.70 ± 13.25	73.06 ± 9.32	0.15
Height (cm)	166.36 ± 8.44	165.88 ± 11.04	0.86
BMI (kg/m^2^)	28.07 ± 4.33	26.66 ± 3.48	0.20
WC (cm)	94.85 ± 9.24	90.87 ± 8.81	0.12
Pemphigus duration (year)	1.71 ± 0.56	1.81 ± 0.60	0.54
Prednisone (mg per day)	9.51 ± 4.19	11.25 ± 5.32	0.19
Disease severity oral *n* (%)			0.27
Quiescent	9 (34.6)	10 (38.5)	
Mild	13 (50)	8 (30.8)	
Moderate	4 (15.4)	8(30.8)	
Severe	0 (0)	0 (0)	
Disease severity skin *n* (%)			0.40
Quiescent	18(69.2)	19 (73.1)	
Mild	5 (19.2)	2 (7.7)	
Moderate	3 (11.5)	5 (19.2)	
Severe	0 (0)	0 (0)	
Physical activity level *n* (%)			0.69
Low	14 (53.8)	11 (42.3)	
Moderate	10 (38.5)	12 (46.2)	
High	2 (7.7)	3 (11.5)	
Medication type *n* (%)			0.73
Prednisone	14 (53.8)	13 (50)	
Prednisone+Me	5 (19.2)	3 (11.5)	
Prednisone+Azathioprine	2 (7.7)	4 (15.4)	
Prednisone+Mycophenolate mofetil	5 (19.2)	6(23.1)	

Abbreviations: BMI, body mass index. WC, waist circumference.

^a^ Variables are expressed as mean ± SD.

^b^ *p*‐values resulted from independent t‐tests for quantitative and chi‐square for qualitative variables between the two groups.

### 3.2 Anthropometric measures and daily dietary intake

Table 1 presents the anthropometric measures and daily dietary intake details of participants throughout the study. No significant differences were observed within and between the two groups in weight, BMI, and dietary intake of total energy (*p* > 0.05). Moreover, results of the ANCOVA test did not show statistically significant differences between the two studied groups in energy, macronutrients and micronutrients intake, adjusted for baseline values (*p* > 0.05). No significant baseline differences between two groups were observed regarding to the type of medications (P > 0.05). There were no significant differences in weight, BMI, WC between placebo and intervention groups.

### 3.3 Biochemical assessment

As shown in Table 2, the independent sample t‐test results revealed no significant differences between the two groups in terms of serum CK, IGF‐1, myostatin, and myogenin levels at baseline (*p* > 0.05). considering that IGF‐1 and CK are affected by age and sex, we analyzed them after age and sex adjusting and the result showed that IGF‐1 level increased and Ck level decreased in the LC group compared to the placebo group. Within‐group comparison showed a significant rise in serum IGF‐1 and reduction in CK. Inter and intra‐group comparison revealed a reduction in serum myostatin level (*p* < 0.05). There was no change in serum myogenin level in the LC group.

**TABLE 2:** Biochemical parameter of muscle metabolism

Variable	l‐carnitine group (*n* = 24)		Placebo group (*n* = 20)		*p* values^a^
	Mean ± SD	Change	Mean ± SD	Change	
WC (cm)					
Baseline	94.85 ± 9.24	− 1.32 ± 3.76	90.87 ± 8.88	− 0.26 ± 1.73	0.35
End of trial	93.51 ± 8.77		90.59 ± 8.94		
*p*‐value	0.08		0.43		
Weight (kg)					
Baseline	77.70 ± 13.25	− 0.63 ± 1.63	73.06 ± 9.32	0.10 ± 2.25	0.25
End of trial	77.06 ± 13.21		73.16 ± 9.27		
*p*‐value	0.058		0.81		
BMI (kg/m^2^)					
Baseline	28.07 ± 4.33		26.66 ± 3.48		0.29
End of trial	27.83 ± 4.29	− 0.23 ± 0.60	26.70 ± 3.37	0.03 ± 0.93	
*p*‐value^b^	0.057		0.83		
IGF‐1					
Baseline	46.74 ± 53.29		49.79 ± 37.41		0.769
After 8 weeks	72.10 ± 37.24	25.36 ± 36.99	58.86 ± 21.90	9.07 ± 43.61	0.172
*p* *	0.06		0.319		
CK					
Baseline	51.36 ± 38.81		49 ± 21.84		0.80
After 8 weeks	41.05 ± 23.27	−10.30 ± 18.65	50.57 ± 19.42	1.57 ± 18.21	0.152
*p* *	0.023		0.682		
Myogenin					
Baseline	136.31 ± 94.11		144.49 ± 71.78		0.749
After 8 weeks	120.77 ± 69.90	−15.54 ± 100.67	67.44 ± 56.06		0.008
*p* *	0.498		0.000		
Myostatin(mmol/L)					
Baseline	6.42 ± 3.7		6.48 ± 16		0.962
After 8 weeks	3.82 ± 1.73	−2.59 ± 3.21	5.89 ± 3.31	−0.59 ± 2.83	0.012
*p* *	0.02		0.315		
Carnitine (nmol/mL)					
Baseline	73.61 ± 34.86	20.28 ± 16.94	77.12 ± 34.33	− 1.73 ± 5.58	<0.001
End of trial	93.89 ± 40.58		75.38 ± 34.59		
*p*‐value^b^	< 0.001		0.12		

Abbreviations: BMI, body mass index. WC, waist circumference. CK, creatine kinase. *p* < 0.05 was considered significant.

* *p* values indicate comparison within groups (paired *t*‐test).

^a^ *p*‐values indicate a comparison between groups (Independent sample *t*‐test at baseline and ANCOVA test).

### 3.4 Discussion

It has been reported that corticosteroid therapy downregulates muscle metabolism and has a muscle‐wasting effect in PV. This was the first study to survey the effects of l‐carnitine supplementation on the muscle‐wasting impact of glucocorticoids in PV patients. l‐carnitine is a dietary supplement with known myotrophic properties and has been reported to have beneficial impact on muscle metabolism. In the present study, LC supplementation led to significant rise in serum IGF‐1 which is one of the main positive regulators of muscle growth. IGF1 is produced by the liver and is associated with protein synthesis. It is a downstream target of growth hormone – increases protein synthesis in skeletal muscle through the Akt–mTOR pathway (Chien et al., 2013). In the present study, inter and intra‐comparison showed a significant rise in level of IGF‐1. The present study confirms recent studies in humans, pigs, chickens, and rats which revealed that supplementation of carnitine increases plasma IGF‐1 concentrations (Beshlawy et al., 2010; Brown et al., 2008; Di Marzio et al., 1999; Doberenz et al., 2006; Heo et al., 2001; Keller et al., 2011; Keller et al., 2013; Kita et al., 2002). Instead, the result of the study in patients with liver cirrhosis showed that carnitine supplementation did not affect the IGF1 levels (Ohara et al., 2018) also Circulating IGF‐I was not affected by LC treatment on porcine fetal (Waylan et al., 2005). This result might be due to the impaired IGF1 production of the liver in these patients.

### 3.5 Myostatin

Myostatin is a member of the transforming growth factor‐b (TGFb) superfamily and an extracellular cytokine that is mostly expressed in skeletal muscles. It inhibits the skeletal muscle mass growth. In the present study, l‐carnitine supplementation led to a significant decrease in serum myostatin levels compared to the placebo group. Recently, Ohara M et al. noted that LC reduced myostatin via decreasing the ammonium level in liver cirrhosis patients (Ohara et al., 2018). However, there is no data about the direct effect of LC on myostatin, various data support the crosstalk between the myostatin and IGF‐1 signaling pathways. IGF‐1 inhibits myostatin signaling through the IGF‐1R/PI3K/Akt pathway; inhibition of the such transcription factors is responsible for the induction of atrogenes via phosphorylation with phosphatidylinositol 3‐kinase (PI3K)/Akt pathway. Akt plays a significant role in different metabolic processes in the cell, particularly in the hypertrophic response to insulin and IGF‐1 (Bodine et al., 2001; Zdychova & Komers, 2005). Therefore, under normal conditions, IGF‐1 signaling seems to be dominant and blocks the myostatin pathway (Trendelenburg et al., 2009) and prevents the TGF‐β family‐mediated apoptosis (Chen et al., 1998). Although most studies have focused on understanding the inhibitory effects of myostatin on IGF‐1 signaling pathway, there is evidence for IGF‐1 interaction with other members of the TGF‐b (Song et al., 2006). Moreover, there are data on how IGF‐1 affects the myostatin signaling pathway (Remy et al., 2004; Retamales et al., 2015). Taken together, LC can reduce the myostatin level through direct pathways like activating the IGF‐1 pathway or decreasing hyperammonemia.

### 3.6 Myogenin

Myogenesis is controlled by a group of transcriptional networks known as the myogenic regulatory factors (MRF). There are four MRFs involved in adult myogenesis, MyoD, Myf5, MRF4, and myogenin. Myogenin is expressed at high levels during the late stages of myoblast differentiation and myotube formation. In the current study after 8 weeks of consumption of the LC, myogenin level was significantly higher in LC compared to placebo, but within the group comparison did not show any changes in the LC group. In line with our study, an in vitro investigation showed that carnitine enhances myotubes differentiation by stimulating the expression of myogenin and skeletal muscle protein myosin heavy chain (Montesano et al., 2015). On the other hand, there is evidence that shows IGF‐1 affects myogenesis positively. One of the molecular mechanisms underlying the stimulatory myogenic effect of IGFs lies in their ability to transcriptionally induce myogenin mRNA (Florini et al., 1991). Moreover, various data noted that myogenin promotor is under control of the IGF/PI3K/Akt pathway (Florini et al., 1991; Quinn et al., 1993; Xu & Wu, 2000). Therefore, there might be an effect of carnitine on myogenin expression through IGF‐1 activation.

### 3.7 Creatine kinase (CK)

CK is a compact enzyme that is found in both the cytosol and mitochondria of tissues where energy demands are high. Raised levels of serum CK are still closely associated with cell damage, muscle cell disruption, or disease. These cellular disturbances can cause CK to leak from cells into the blood serum. Measurement of serum CK activity and determination of isoenzyme profiles are important indicators of occurrence of muscle cell necrosis and tissue damage. Serum CK is the most diagnostically sensitive test for muscle injury and myopathies (Clarkson et al., 2006; Hoffman & Clemens, 1996; Mirzazadeh et al., 2014). In this study, serum CK level was significantly lower in the LC group compared to the control group. In addition, within‐group comparisons showed that the level of CK significantly decreased (*p* = 0.06) after 8 weeks of LC supplementation. Along with our result, in a cross‐over study, Giamberardino et al. (1996)) showed that supplementation with l‐carnitine alleviated pain and release of creatine kinase. Moreover, daily intake of 2 g l‐carnitine compared to placebo accompanied by a significant reduction in released cytosolic proteins such as creatine kinase (Spiering et al., 2008; Volek et al., 2002). In contrast, another clinical trial by Mariano Malaguarnera and colleagues in centenarians' serum, showed no significant change in Ck level (Malaguarnera et al., 2007). This discrepancy between our findings with previous studies might be due to the short duration of intervention which could not affect the CK enzyme activity to have a significant change between the two groups. The limitations of the present study that need to be considered in interpreting the results included the short duration of the intervention and small sample size. The strength of our study was monitoring patient status through weekly telephone conversations and serum LC levels of the participants during the trial were measured to evaluate their adherence to the LC supplementation. Moreover, l‐carnitine appeared to be well tolerated by participants and anecdotal reports indicated that the intervention was acceptable to them.

## 4 CONCLUSION

In conclusion, these observations support the myotrophic role of carnitine which is most likely due to the IGF‐1 signaling pathway on muscle protein synthesis and prevents apoptosis. Myogenin is a good marker for muscle growth but it needs a muscle biopsy to reveal the exact result. Moreover, the result of this study supports the inhibitory effect of LC on myostatin. Therefore, LC supplementation increases the serum IGF‐1 and changes the myostatin, myogenin level, and CK activity. However, the mechanism for this effect of LC is not completely elucidated, IGF‐1 is one of the molecules which mediates the LC impact on myostatin and myogenin.

### AUTHOR CONTRIBUTION

Zeinab Noormohammadi: Data collection, analysis and interpretation of results, draft manuscript preparation, paper writing. Hana Arghavani: Data collection, interpretation of results. Mohammadhasan Javanbakht: Study conception and design, analysis, and interpretation of results. Maryam Daneshpazhooh: Study conception and design. Mahmood Jalali: Study conception and design.

### CONFLICT OF INTEREST STATEMENT

The authors declare no conflict of interest.
