# Correction to “L‐carnitine downregulate the muscle wasting effect of glucocorticoids in pemphigus patient: A randomized double‐blind placebo‐controlled study”

**DOI:** 10.14814/phy2.15738

**Published:** 2023-06-07

Noormohammadi, Z., Arghavani, H., Javanbakht, M., Daneshpazhooh, M., and Jalali, M. (2023). L‐carnitine downregulate the muscle wasting effect of glucocorticoids in pemphigus patient: A randomized double‐blind placebo‐controlled study. *Physiological Reports*, 11, DOI: 10.14814/phy2.15657.

In the above article, the fifth author's name was spelled incorrectly. The correct spelling is M. Jalali

The affiliation of the fourth author was also incorrect. The correct affiliation is

Autoimmune Bullous Diseases Research Center, Department of Dermatology team, Tehran University of Medical Sciences, Tehran, Iran

These errors have also been corrected online.

We apologize for this error.

